# Detection of circulating tumor cells in blood of pancreatic ductal adenocarcinoma patients

**DOI:** 10.20517/cdr.2019.73

**Published:** 2020-03-19

**Authors:** Andrea Mayado, Alberto Orfao, Anouk Mentink, Maria Laura Gutierrez, Luis Muñoz-Bellvis, Leon W.M.M. Terstappen

**Affiliations:** ^1^Cancer Research Center (IBMCC-CSIC/USAL-IBSAL), Cytometry Service (NUCLEUS) and Department of Medicine, Universidad de Salamanca, Salamanca 37007, Spain.; ^2^Centro de Investigación Biomédica en Red de Cáncer (CIBER-ONC) CB16/12/00400, Instituto de Salud Carlos III, Madrid 28029, Spain.; ^3^Department of Medical Cell BioPhysics, University of Twente, Enschede 7522, The Netherlands.; ^4^Division of General and Gastrointestinal Surgery, University Hospital of Salamanca (CAUSA), and Department of Surgery, Universidad de Salamanca and IBSAL, Salamanca 37007, Spain.

**Keywords:** Pancreatic ductal adenocarcinoma, circulating biomarkers, circulating tumor cells

## Abstract

**Aim:** Previous studies suggest that circulating tumor cells (CTC) are present at very low frequencies in blood of pancreatic cancer (PC) patients. However, no technique has proven efficient for their detection, in part due to the lack of accurate tumor markers. Here, we evaluated the potential utility of two marker candidates - Mucin 16 (MUC16) and Tetraspanin 1 (TSPAN1) - identified through a detailed review of the literature.

**Methods:** To evaluate the pattern of expression of both markers in pancreatic tumor cells *vs*. normal blood, we used cell lines derived from pancreatic cancer patients and blood from healthy adults.

**Results:** Antibodies against both MUC16 and TSPAN1 showed expression in three pancreatic cancer (PC) cell lines while they were absent in blood cells. To evaluate the efficiency of isolating tumor cells from blood, PC cell lines were spiked at different frequencies in blood, sequentially stained with biotin-conjugated TSPAN1 and MUC16 antibodies and a streptavidin ferrofluids, followed by immunomagnetic enrichment. The recovery of spiked TSPAN1^+^ tumor cells was high with limited contamination by leukocytes. In contrast, no PC cells were isolated when the biotin MUC16 reagent was used because the biotin-conjugated clone did not recognize PC cells.

**Conclusion:** The combination of MUC16, TSPAN1, and epithelial cell adhesion molecule (EpCAM) antibodies will likely increase the efficiency of capturing circulating tumor cell in blood of pancreatic ductal adenocarcinoma. To further develop a protocol for isolation of circulating tumor cell in blood of PC patients, high amounts of antibodies (5-10 mg) against EpCAM, MUC16, and TSPAN1 will be needed.

## Introduction

Pancreatic ductal adenocarcinoma (PDAC) represents ~3% of all newly-diagnosed cancer patients^[1]^ (10.5 cases per 100,000/year in the EU). Despite this, it represents the fourth cause of death in the western world due to its very poor prognosis, with a five-year survival rate of 5%^[2-5]^, mainly caused by delayed diagnosis and resistance to conventional therapy. Therefore, early diagnosis of PDAC is key to improving the outcome of this poor prognosis cancer type. Currently, diagnosis of PDAC is primarily triggered by unspecific symptoms/signs of pancreatic disease that emerge at relatively advanced disease stages, followed by low-sensitive imaging techniques and histopathology of suspicious lesions^[6]^. The potential utility of serum biomarkers such as carbohydrate antigen 19-9 (CA19-9) for the diagnostic screening of PDAC has been extensively evaluated. However, these markers (e.g., CA19-9) lack sensitivity and specificity for early PDAC detection with a significant percentage of both false negative and false positive results. Thus, current use of serum biomarkers such as CA19-9 is restricted to monitoring response to therapy among patients who presented with elevated levels at diagnosis. Similarly, different combinations of CA19-9 with multiple other serum biomarkers [e.g., laminin subunit gamma 2 (LAMC2), carcinoembryonic antigen (CEA), insulin-like growth factor 1 (IGF-1), intercellular adhesion molecule-1 (ICAM-1), osteoprotegerin (OPG), C - reactive protein (CRP), interleukin (IL), platelet-derived growth factor (PDGF)] have also been evaluated^[7]^, but failed to provide a reliable PDAC screening tool. Thereby, robust (sensitive and specific), cost-effective markrs are still required for early (minimally-invasive) diagnosis of PDAC that would lead to early treatment and improved PDAC patient outcome.

In the last two decades, detection of circulating tumor cells (CTC) in blood and tumor-associated genetic biomarkers in plasma has emerged as promising sensitive diagnostic approaches. Even though PDAC cells are primarily located in the pancreas and its metastatic sites, CTC can also be detected at very low frequencies in peripheral blood (PB) in at least a fraction of all PDAC patients^[8]^. CTC techniques have proven to be easy to perform, minimally invasive, and accurate in detecting cancer cells. However, some technical limitations of CTC detection methods still exist, particularly as regards the most informative marker for the identification of circulating PDAC cells and their subsequent isolation. Among other markers, several members of the tetraspanin family of adhesion molecules, such as tetraspanin 1 (TSPAN1)^[9]^ and mucins (a family of high molecular weight and heavily glycosylated proteins, known to play an important role in the pathogenesis of PDAC)^[10]^, particularly mucin 16 (MUC16) involved in metabolic reprogramming of pancreatic cancer cells via its effects on an increased glycolysis and enhanced motility and invasiveness of PDAC tumor cells^[11]^, are candidate PDAC-associated protein markers. However, the utility of these markers for the detection and isolation of circulating PDAC tumor cells in blood still needs to be demonstrated. TSPAN1 has recently been demonstrated to be elevated in human primary PDAC tumor cells and cell lines, in addition to high-grade cervical intraepithelial neoplasia and advanced cervix carcinoma^[12]^, lung cancer^[13]^, colon cancer^[14]^, breast cancer^[15]^, and squamous cell carcinoma^[16]^. Similarly, Gutierrez *et al*.^[17]^ observed increased mRNA expression of GPR137B, S100A11, sulfatase (SULF1), and periostin (POSTN) in PDAC *vs*. normal pancreatic tissues, but how this translates into protein expression remains to be demonstrated.

Here, we developed a PDAC-oriented approach for diagnosis and monitoring of PDAC patients, based on detection and positive selection of blood cells expressing the epithelial cell adhesion molecule (EpCAM) compared to other protein biomarkers (MUC16 and TSPAN1) that might increase the efficiency of capturing circulating PDAC tumor cells in blood.

## Methods

### Patients, healthy donors, and samples

Nine consecutive PB samples from PDAC patients (67% males and 33% females; median age of 70 years, ranging from 44 to 83 years), collected from November 2019 to February 2019 at the Department of Surgery of the University Hospital of Salamanca (Salamanca, Spain), were included in this study [Table 1]. In parallel, normal PB samples from five (anonymized) healthy volunteers obtained through the TNW-ECTM-donor services of the University of Twente (Enschede, The Netherlands) were also studied. In every case, PB samples were drawn by venipuncture into 10 mL CellSave collection tubes (Menarini Silicon Biosystems, Huntingdon Valley, PA) or vacutainer tubes containing EDTA as anticoagulant [Becton/Dickinson (BD), Franklin Lakes NJ].The study (in case of patients) and blood collection (in case of healthy donors) were approved by the local ethics committees of the University Hospital of Salamanca (Salamanca, Spain) and the University of Twente (Enschede, The Netherlands), respectively, and the research complied with all applicable laws and institutional guidelines. Informed consent was given by each individual prior to entering the study, according to the Declaration of Helsinki.

**Table 1:** Patient characteristics at diagnosis

Patient code	Diagnosis	Age	Sex	Metastasic sites	Tumor stage (TNM)	Surgical resection
1	PDAC	70	Male	-	IA (T1N0M0)	R0
2	PDAC	82	Female	Liver	IV	R1
3	PDAC	73	Male	NA	IV	R1
4	PDAC	83	Female	Liver	IV	R1
5	PDAC	44	Male	Liver	IV	R1
6	PDAC	63	Female	Mesentery, peritoneum	IV	R1
7	PDAC*	63	Male	No findings	NA	R1
8	Resectable PDAC	58	Male	-	IIA (T3N1M0)	R0
9	PDAC	70	Male	-	IA (T1N0M0)	R0

*Patient analyzed after neoadjuvant therapy. NA: not available; R0: microscopically negative resection margins; R1: microscopically positive resection margins; PDAC: pancreatic ductal adenocarcinoma

### Pancreatic cell lines

To evaluate antibody expression profiles, the CAPAN-1, CAPAN-2, and MIA PaCa-2 cell lines were used. Briefly, CAPAN-1 cells (median size of 16.5 μm) were obtained from the American Type Culture Collection (ATCC; Manassas, VA) and grown at 37 °C in Iscove’s Modified Dulbecco’s Medium (IMDM; Sigma-Aldrich, St. Louis, MO) containing 2 mM *L*-glutamine (G7513, Sigma-Aldrich) supplemented with 10% fetal bovine serum (FBS; Invitrogen, Thermo Fisher Scientific, Inc., Waltham, MA) and 1% (v/v) penicillin/streptomycin (Invitrogen), in a humidified atmosphere containing 5% CO_2_. Cells were passaged until they reached 70%-80% confluence, detached using 0.25% Trypsin-EDTA (1X) with Phenol Red (Gibco, Thermo Fisher Scientific, Inc.), and replated at a seeding density > 25, 000 cells/cm^2^. Culture medium was refreshed twice a week and cells counted using the Luna automated cell counting system (Logos Biosystems, Annandale, VA) by loading 12 μL of the cell suspension into the corresponding counting slide. In turn, CAPAN-2 cells (median size of 17.9 μm) were grown in RPMI 1640 medium (Lonza Group Ltd, Basel, Switzerland) containing 2 mM *L*-glutamine supplemented with 15% FBS and 1% (v/v) penicillin/streptomycin under identical culture conditions as described above for CAPAN-1 cells. MIA PaCa-2 cells (median size of 16.2 μm) were cultured in Dulbecco’s modified Eagle’s medium (DMEM; HyClone; GE Healthcare, Logan, UT) containing 2 mM *L*-glutamine supplemented with 10% FBS (v/v) and 1% (v/v) penicillin/streptomycin, and processed as previously described in this section for CAPAN-1 cells.

### Selection of PDAC-associated target proteins

The following criteria based on review of the literature were used to select for candidate PDAC-associated target proteins: (1) up-regulated gene expression in PDAC *vs*. normal pancreatic tissues with concordant gene *vs*. protein expression patterns in PDAC; (2) normal pancreatic tissue-specific proteins which are not significantly downregulated in PDAC; (3) proteins expressed in the cell surface membrane; and (4) proteins expressed in both primary PDAC tumor cells and pancreatic cancer cell lines according to the Human Protein Atlas (www.proteinatlas.org). Based on the above criteria, MUC16 and TSPAN1 were selected to be evaluated as target PDAC-associated proteins [Table 2].

**Table 2:** General features and tissue distribution of the MUC16 and TSPAN1 proteins and the corresponding antibody reagents used in this study

Protein/Antibody features	Mucin 16 (MUC16/CA125)^[10]^	TSPAN1^[9,18,19]^
Tissue distribution Cancer tissues		
Colon	-	+
Esophageal	-	+
Gastric	-	+
Liver	-	+
Ovarian cancer	+	+
Pancreatic	-	+
Normal tissues	Cervix, uterine and fallopian tube	Colon and rectum
Cancer cell lines	CAPAN-2	CAPAN-2
Cell surface membrane	+	+
Evidence at the protein level	+	+
Monoclonal antibody clone (source) reagent	Clone #986808 (R&D systems)	Clone #819202 (Novus Biologicals)
Biotin-conjugated antibody (source)	X306 (Gene Tex)	Polyclonal (Abbexa)

TSPAN1: tetraspanin 1; MUC16: mucin 16

### Evaluation of MUC16, TSPAN1 and EpCAM expression on PDAC cell lines and PB cells

Briefly, 200,000 cells from each cell line or 100 µL PB were incubated with 5 µg/mL of the anti-MUC16 antibody (clone #986808 from R&D systems, McKinley Place, MN), the biotin-MUC16 antibody reagent (clone X306) (Gene Tex, Hsinchu City, Taiwan), the TSPAN1 antibody (clone #819202) (Novus Biologicals, Centennial, CO), the biotin-TSPAN1 (polyclonal) antibody reagent (Abbexa, Cambridge, UK), or 2.5 µg/mL of anti-EpCAM antibody (30 min at 37 °C). Unconjugated and biotinylated antibodies were used to allow for a brighter (i.e., amplified) and quantifiable (comparable among antibody reagents) fluorescence signal due to signal in the flow cytometer. After two washes with phosphate-buffered saline (PBS) containing 1% bovine serum albumin (BSA) (Sigma-Aldrich), stained cells were incubated with an anti-mouse IgG-phycoerythrin (PE) antibody (Sigma-Aldrich) in case of cells stained with primary unconjugated antibodies or a streptavidin-PE (Sigma-Aldrich) reagent for cells stained with the biotin reagents, for another 30 min at 37 °C. After two washes, cells were resuspended in PBS containing 1% BSA, and MUC16, TSPAN1, and EpCAM expression were measured in a FACS ARIA II flow cytometer (BD Biosciences, San Jose, CA). In the case of PB, a lysing step was performed, consisting of a stain-lyse-and-wash protocol that uses FACS Lysing solution (BD Biosciences, San Jose, CA), as described elsewhere^[20]^.

### Spike-in of CAPAN-2 and MIA PaCa-2 cells in normal PB

Approximately 3000 cells pre-stained with 10 μM of Cell Tracker Green (Life Technologies Corporation, Carlsbad, CA) of each cell line (TRUCOUNT tubes, BD) were spiked in separate tubes containing 1 mL of normal PB each. Subsequently, samples were incubated for 15 min at room temperature (RT) with 1 µg/mL of biotin-EpCAM with or without 5 µg/mL biotin-TSPAN1. Afterward, 4 mL of Dilution Buffer (Menarini Silicon Biosystems Inc, Bologna, Italy) was added to each tube, followed by a centrifugation step for 10 min at 800 *g*. After removing the supernatant, 3 mL of Dilution Buffer was added and samples incubated with 40 µg/mL of streptavidin-ferrofluids (Biomagnetic Solutions, State College, PA) for 15 min at RT. The cell solution was gently mixed and placed into a quadrupole magnet for 20 min. The tumor cell-enriched fraction was gently collected by aspiration and washed in PBS/BSA 1%. Subsequently, each sample was divided into three aliquots: one was directly measured in a flow cytometer, while the second was incubated with streptavidin-PE and the third with an anti-mouse IgG-PE antibody, prior to measurement in the flow cytometer. All cell solutions were stained with 4 μg/mL of Hoechst 33342 in PBS (Life Technologies Co). The actual number of EpCAM, TSPAN1, and MUC16 protein molecules/cell was determined using QuantiBRITE beads (BD Biosciences).

### Immunomagnetic CTC enrichment and filtration

Approximately 250 cells from each cell line pre-stained with 10 μM Cell Tracker Green were spiked each in 7.5 mL of PB from three healthy volunteers. Samples were then incubated for 15 min at RT with 1 µg/mL of biotin-EpCAM and 5 µg/mL biotin-TSPAN1. After this incubation, 5.5 mL of Dilution Buffer was added, and the sample centrifuged for 10 min at 800 *g*. Subsequently, the supernatant was removed, 4.5 mL of Dilution Buffer added to the sample, and another incubation with 40 µg/mL of streptavidin-ferrofluids was performed for 15 min at RT. Afterward, the sample was gently mixed and placed into a quadrupole magnet for 20 min. The fraction enriched on CTC and the unbound cell fraction were both collected and washed with 15 mL of PBS/BSA 1%.

To filter tumor cells from both the enriched and the unbound cells fractions, microsieves were used (VyCAP, Deventer, The Netherlands). Each microsieve contains 111,800 pores of 5 μm diameter spaced 14 μm apart in lanes with a porosity of 10%, on a total surface area of 8 mm × 8 mm. The microsieve was contained in a plastic holder placed in a disposable filtration unit. The enriched and unbound cell fractions were transferred to separate microsieves and filtration units. The filtration units were then placed on a pump unit that maintained a pressure of < 10^5^ mbar across the microsieve during filtration (VyCAP). At the end of the filtration process, any remaining unfiltered sample volume was removed with a pipette. Next, the microsieve was removed and placed in the staining holder, washed with PBS/BSA 1% and incubated for 20 min at RT with a fixation buffer - 100 µL of solution A of the FIX & PERM reagent KIT (Nordic MUbio, Susteren, The Netherlands) plus 50 µL of PBS/BSA 1%. Once fixed, the filtered cells in the microsieve were permeabilizated and stained with a solution containing: 0.5 µL of anti-pan Cytokeratin (CKs 1-8, 10, 14, 15, 16, and 19) antibody (clone AE1/AE3) (eBioscience Inc, San Diego, CA) conjugated with PE, 4 µL of anti-C11 (clone# 6030V LN: E944) (Veridex, Raritan NJ) conjugated with PE, 2 µL of anti-CD45 PerCP (Life Technologies Co), and 43.5 µL of solution B of the FIX & PERM reagent KIT for 20 min at RT. Afterward, the microsieve was washed and incubated for 5 min (RT) with PBS containing 1% BSA. Removal of the fluid during each of the staining and washing steps was done by bringing the bottom of the microsieve into contact with an absorbing material in the staining holder (VyCAP). The microsieve was subsequently covered with ProLong® Diamond Antifade Mountant containing 4’,6-diamidino-2-phenylindole (DAPI) (Thermo Fisher Scientific). A custom cut 0.85 cm × 0.85 cm glass cover slip (Menzel-Gläser, Saarbrükener, Germany) was placed on both sides of the microsieve for immediate analysis or storage at 4 °C until analyzed. An identical immunomagnetic CTC-enrichment followed by filtration was also applied for detection of CTC in blood of PDAC patients (*n* = 9). Fluorescent images from the microsieves were captured and analyzed for identification of CTC using the ICY open-source software available from http://icy.bioimageanalysis.org/. Operators were asked to annotate every DAPI^+^ CK^+^ CD45- event and classify the event as a CTC when morphological features were consistent with that of a cell.

## Results

### TSPAN1 expression on CAPAN-1, CAPAN-2 and MIA PaCa-2 cells

**Figure 1:**
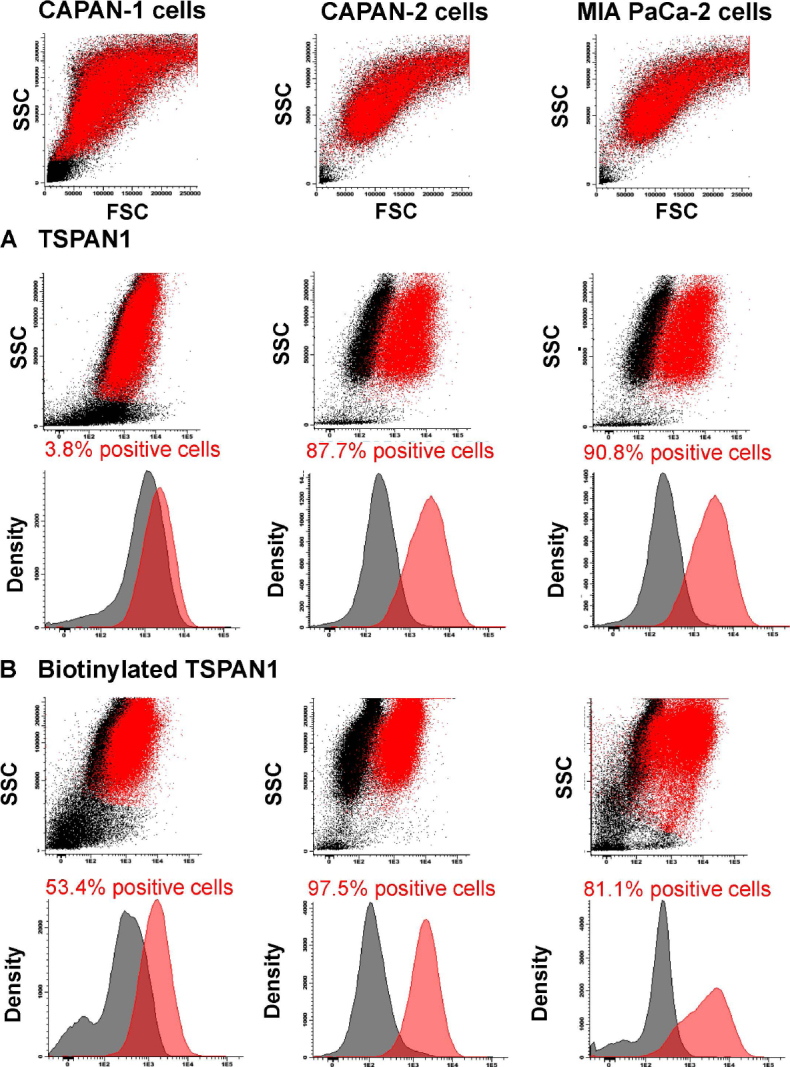
TSPAN1 expression observed for the CAPAN-1, CAPAN-2 and MIA PaCa-2 cell lines. Staining with unconjugated (A) or biotinylated (B) anti-TSPAN1 antibody (5 µg/mL) reagents (red dots and histograms) compared to a negative control staining (black dots and histograms) is shown. Flow cytometry dot plots and histograms correspond to merged flow cytometry data files of sample aliquots prepared under identical conditions with or without the TSPAN1 antibody. TSPAN1: tetraspanin 1; SSC: side scatter; FSC: forward scatter

**Figure 2:**
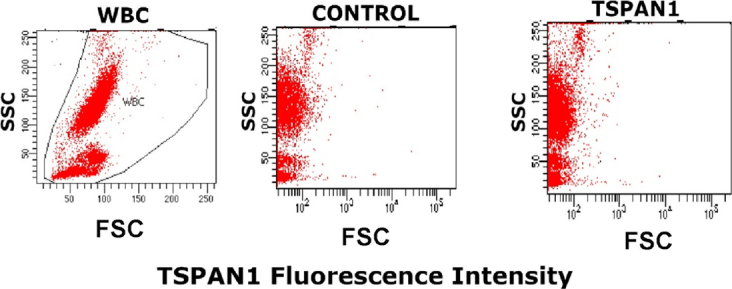
TSPAN1 expression on normal white blood cells. WBC size *vs.* complexity representation (left).The staining profile of a healthy adult blood sample for the unconjugated anti-TSPAN1 (5 μg/mL) antibody (right) compared to a control aliquot of the same sample prepared under identical conditions except that it was not stained with the for the anti-TSPAN1 antibody reagent (middle). TSPAN1: tetraspanin 1; WBC: white blood cells; SSC: side scatter; FSC: forward scatter

Overall, TSPAN1 was found to be unequivocally expressed on CAPAN-2 and MIA PaCa-2 cells when both unconjugated (87.7% and 90.8% positive cells, respectively) [Figure 1A] and biotinylated reagents (97.5% and 81.1% positive cells, respectively) [Figure 1B] were used. In contrast, CAPAN-1 cells showed low TSPAN1 expression (3.8% positive cells) compared with the other two PDAC cell lines [Figure 1A and B]. In turn, TSPAN1 expression was absent in virtually all white blood cells (WBC) in blood of healthy adults tested with the non-biotinylated (unconjugated) TSPAN1 antibody [Figure 2].

### MUC16 expression on PDAC cell lines

**Figure 3:**
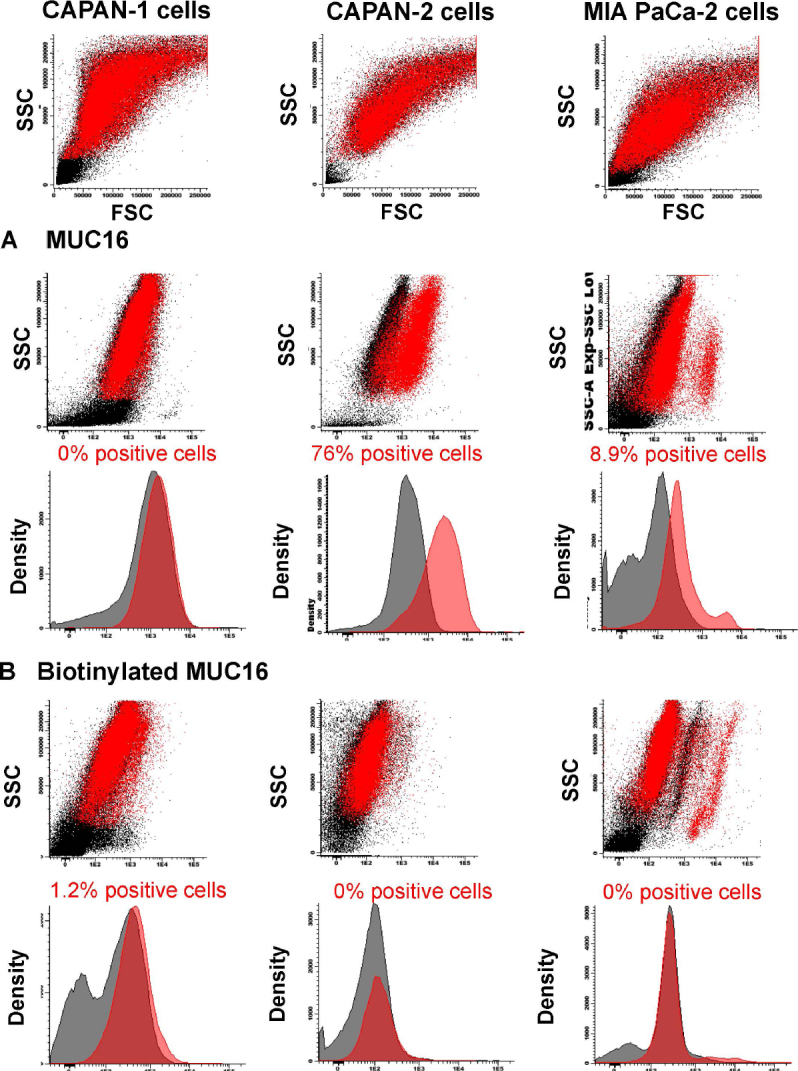
MUC16 expression observed for the CAPAN-1, CAPAN-2 and MIA PaCa-2 cell lines. Staining with unconjugated (A) or biotinylated (B) anti-MUC16 antibody (5 µg/mL) reagents (red dots and histograms) compared to a negative control staining (black dots and histograms). Flow cytometry dot plots and histograms correspond to merged flow cytometry data files of sample aliquots prepared under identical conditions with or without the MUC16 antibody. MUC16: mucin 16; SSC: side scatter; FSC: forward scatter

**Figure 4:**
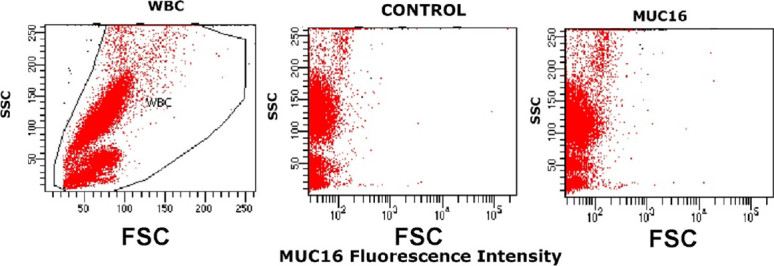
MUC16 expression on normal white blood cells. WBC size *vs.* complexity representation (left). An example of the staining observed for a normal PB sample staining with an unconjugated anti-MUC16 (5 μg/mL) antibody (right) and the same sample processed in parallel under the same conditions but without anti-MUC16 reagent (middle). MUC16: mucin 16; WBC: white blood cells; SSC: side scatter; FSC: forward scatter

No MUC16 expression was found on CAPAN-1, while clear MUC16 staining was observed for the great majority of CAPAN-2 cells (76% of positive cells) and a minor subset of MIA PaCa-2 cells (8.9% of positive cells) [Figure 3A] with the unconjugated anti-MUC16 antibody reagent, but not with the biotinylated antibody clone [Figure 3B]. As found for TSPAN1, MUC16 was also absent on normal blood leucocytes (stained with the non-biotinylated antibody reagent) [Figure 4].

### EpCAM expression on PDAC cell lines

**Figure 5:**
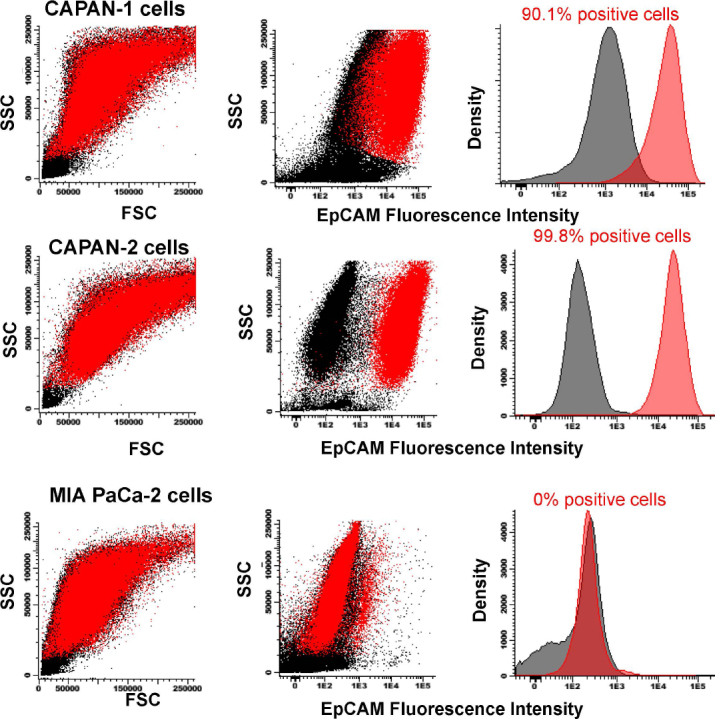
EpCAM expression on CAPAN-1, CAPAN-2 and MIA PaCa-2 cells. Staining with unconjugated anti-EpCAM (2.5 µg/mL) (red dots and histograms) *vs*. control samples (black dots and histograms). Flow cytometry dot plots and histograms correspond to merged flow cytometry data files prepared under identical conditions with or without the EpCAM antibody. EpCAM: epithelial cell adhesion molecule; SSC: side scatter; FSC: forward scatter

Overall, EpCAM was found to be expressed on CAPAN-1 (90.1%) and CAPAN-2 (99.8%) cells [Figure 5]. In contrast, MIA PaCa-2 cells showed no EpCAM expression with fluorescence levels similar to those of the control samples processed under the same conditions but without the anti-EpCAM antibody reagent [Figure 5].

### Level of expression of EpCAM, TSPAN1 and MUC16 protein molecules per cell

In line with the above findings, CAPAN-2 cells showed the highest amounts of EpCAM, TSPAN1, and MUC16 levels per cell [Table 3], while MIA PaCa-2 cells showed slightly lower levels of TSPAN1 in the absence of EpCAM and MUC16 expression. In turn, CAPAN-1 cells showed the highest levels of expression for EpCAM associated with low amounts of TSPAN1 expressed per cell in the absence of MUC16 in the great majority of the cells (please see text above).

**Table 3:** Amount of EpCAM, TSPAN1, and MUC16 protein molecules expressed per cell on the surface of CAPAN-1, CAPAN-2, and MIA PaCa-2 cells

Cell line	Antibody bound per cell (unstained)	EpCAM	TSPAN1	MUC16
CAPAN-1	16,125.6	327,927	12,179	2484
CAPAN-2	2179.8	252,073	27,526	19,303
MIA PaCa-2	2554.1	-	23,578	-

Results expressed as antibody binding capacity per cell evaluated with Quantibrite phycoerythrin beads. TSPAN1: tetraspanin 1; MUC16: mucin 16; EpCAM: epithelial cell adhesion molecule

### Recovery of spiked tumor cells in normal blood

To simulate the isolation of blood CTC in cancer patients, CAPAN-2 or MIA PaCa-2 cells pre-stained with either the EpCAM or the EpCAM plus the TSPAN1 antibodies were spiked at known numbers in 1 mL of PB samples aliquots from two healthy volunteers. In one healthy donor, the combination of EpCAM and TSPAN1 was associated with a better recovery of the spiked cells compared to EpCAM alone with a percentage recovery of 80% *vs*. 35% for CAPAN-2 cells and of 38% *vs*. 18% for MIA PaCa-2 cells [Table 4]. In the second healthy control, simple recovery of CAPAN-2 was also good with EpCAM plus TSPAN1 staining (mean 63% ± 18%) particularly when Streptavidin-ferrofluids (75%) or Streptavidin-PE (71%) was used as secondary antibody reagents [Table 4]. These preliminary results suggest that the combination of EpCAM and TSPAN1 could help increase the recovery of isolated pancreatic tumor cells from pancreatic cancer patient’s blood.

**Table 4:** Recovery of CAPAN-2 and MIA PaCa-2 cells spiked in normal peripheral blood of two healthy donors based on immunomagnetic isolation of EpCAM *vs*. EpCAM plus TSPAN1 stained cells

Blood samples	*n* of spiked cells	Antibodies	*n* of recovered cells (%)	Mean recovery of duplicates
Donor 1	3000 CAPAN-2	EpCAM	1089 (36%)	1043 (35%)
			996 (33%)	
		EpCAM + TSPAN1	1672 (56%)	2397 (80%)
			3122 (100%)	
	3000 MIA PaCa-2	EpCAM	593 (20%)	521 (18%)
			448 (16%)	
		EpCAM + TSPAN1	1321 (44%)	1155 (38%)
			988 (32%)	
Donor 2	3000 CAPAN-2	EpCAM +TSPAN1 Streptavidin-FF	2414 (81%)	225 (75%)
			2100 (70%)	
		EpCAM + TSPAN1 Streptavidin-PE	2076 (69%)	2127 (71%)
			2178 (73%)	
		EpCAM + TSPAN1 Anti-mouse-PE	1390 (46%)	1267 (42%)
			1144 (38%)	

FF: ferrofluids; PE: phycoerythrin; EpCAM: epithelial cell adhesion molecule; TSPAN1: tetraspanin 1

### Validation of the immunomagnetic CTC enrichment protocol followed by filtration

**Figure 6:**
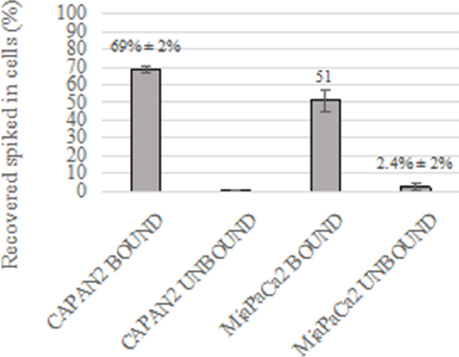
Recovery of CAPAN-2 and MIA PaCa-2 cells spiked in normal peripheral blood and stained simultaneously with anti-TSPAN1 and anti-EpCAM antibodies. EpCAM: epithelial cell adhesion molecule; TSPAN1: tetraspanin 1

Approximately 250 CAPAN-2 cells or 250 MIA PaCa-2 were spiked in 7.5 mL of PB from three healthy donors to validate the specificity of the immunomagnetic CTC-enrichment protocol. Thus, by sequential immunomagnetic enrichment with TSPAN1 and EpCAM followed by filtration (as described above in the Methods Section), relatively high recovery rates of stained CAPAN-2 and MIA PaCa-2 cells were achieved [Figure 6 and Table 5]. In line with the results described in the previous section, recovery of CAPAN-2 cells from the CTC-enriched cell fraction was high (median of 69% ± 2%) and rather stable for all three samples analyzed (range: 67%-71%) [Figure 6 and Table 5]. In turn, for MIA PaCa-2 cells, the overall recovery was lower than for the CAPAN-2 cells (mean 51% ± 6%) ranging between 44% and 56% for the three samples tested. Note that recovery of CAPAN-2 and MIA PaCa-2 cells from the unbound CTC-depleted fractions was almost negligible (mean 0.1% ± 0.2% and 2.4% ± 2%, respectively) [Figure 6].

**Table 5:** Recovery of CAPAN-2 cells or MIA PaCa-2 cells spiked in blood of healthy donors (*n* = 3) after immunomagnetic CTC enrichment with the anti-TSPAN1 and anti-EpCAM antibodies and large cell filtration

Blood samples	*n* of spiked cells (cell line)	CTC-enriched cell fraction	CTC-depleted cell fraction
Sample 1	307 (CAPAN-2)	217 (71%)	1 (0%)
	270 (MIA PaCa-2)	151 (56%)	3 (1%)
Sample 2	209 (CAPAN-2)	145 (69%)	0 (0%)
	294 (MIA PaCa-2)	157 (53%)	13 (4%)
Sample 3	196 (CAPAN-2)	131 (67%)	0 (0%)
	197 (MIA PaCa-2)	87 (44%)	3 (2%)

CTC: circulating tumor cell; TSPAN1: tetraspanin 1; EpCAM: epithelial cell adhesion molecule

### CTC detection and isolation from PDAC patient’s blood

**Figure 7:**
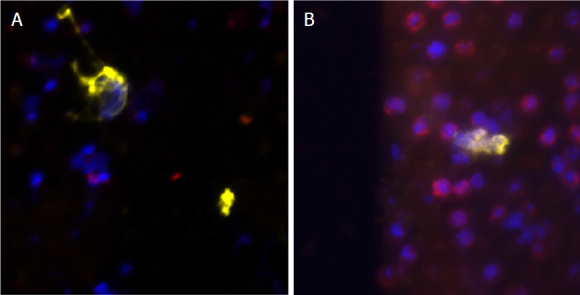
The microphotographs of CTC detected in the two CTC+ PDAC patients (A and B). Cytokeratin staining is shown in yellow, DAPI-stained nuclei are depicted in blue, and CD45 staining is in red. CTC: circulating tumor cell; PDAC: pancreatic ductal adenocarcinoma

CTC detection and enumeration in 7.5 mL of blood based on EpCAM plus TSPAN1 staining followed by CTC-enrichment and filtration was performed in nine PDAC patients. The criteria for a cell to be identified as a CTC were as follows: nucleated (DAPI^+^) intact cells, positive for pan-cytokeratin 8, 18, and 19 (EpCAM^+^), negative for CD45, and a well-defined tumor cell-like morphology. Overall, CTC were found in two (22%) of nine patients tested (Cases #2 and #6 in Table 1), as illustrated in Figure 7. CD45^+^ EpCAM^+^ cells were found in no patients.

## Discussion

PDAC remains one of the most devastating diseases because of delayed late diagnosis and high frequency of deaths due to, e.g., metastatic and invasive disease. Currently available biopsy strategies and scanning/imaging technologies do not provide the desired sensitivity and specificity for early diagnosis of PDAC, mostly because they are time-consuming and/or invasive procedures not suitable for PDAC screening. Therefore, an urgent need exists for novel biomarkers for early diagnosis of PDAC. In recent years, detection and characterization of CTC has become feasible and of proven great clinical utility in distinct types of cancer due to the high-sensitivity reached by novel CTC detection methods, the minimally invasive nature of the blood-based procedure, and its correlation with tumor diagnosis and patient outcome. Since their discovery, CTC are identified for the expression of CK 8/18/19 and lack of CD45 expression (EpCAM^+^ CK^+^ CD45- cells)^[21]^. However, CTC data in PDAC still rely to a large extent on small patient cohorts at various stages of the disease based on distinct CTC techniques. Despite this, such studies indicate that presence of CTC in blood of around one third of PDAC patients (34%) who showed (significantly) poorer survival^[22,23]^. In addition, presence of CTC in PDAC has been associated with poorly-differentiated tumors and occult metastatic disease prior to surgery^[23]^. A more detailed analysis of the most representative studies shows disturbing levels of variability in the frequency of CTC^+^ PDAC patients associated with the use of distinct CTC-detection approaches including techniques based on CellSearch (Silicon Biosystems) technology and Nano Velcro assays, among other approaches. In addition, different methods (e.g., density, electric charges, and deformability) and features (e.g., size) have also been used for isolation of suspicious cells and demonstration of their CTC nature^[24]^.

Among other cell membrane markers, EpCAM has been the most frequently used. However, although most pancreatic tumors are EpCAM positive (96%)^[25]^, EpCAM expression levels in PDAC cells are heterogeneous with only half of tumors showing strong expression in reasonable percentages of the tumor cells^[26,27]^. Altogether, these results led us to investigate the potential value of other markers for improving CTC detection in blood of PDAC patients.

Based on prior research of the available literature, two markers, in addition to EpCAM, were studied: MUC16 and TSPAN1. Evaluation of the expression profiles for both markers on the CAPAN-1, CAPAN-2, and MIA PaCa-2 cell lines showed that TSPAN1 was strongly expressed in both CAPAN-2 and MIA PaCa-2 but not CAPAN-1 cells, while it was found to be absent in WBC. In contrast, MUC16 was not found to be present in CAPAN-1, CAPAN-2, and MIA PaCa-2 cells once tested with a biotinylated anti-MUC16 antibody reagent, while it was expressed in most CAPAN-1 and a minor fraction of MIA PaCa-2 cells when the prior antibody was replaced by a distinct (unconjugated) anti-MUC16 antibody clone. Altogether, these results suggest that TSPAN1 could be a useful biomarker for detection of CTC in PDAC patients, particularly when combined with EpCAM, while further evaluation of different antibody clones is required to identify an optimal reagent for the identification and subsequent isolation of CTC in these patients. Note that TSPAN1 has been shown to play an important role in human pancreatic cancer cell migration and invasion, through modulation of the expression of the matrix metalloproteinase 2 (MMP2) via phospholipase Cγ^[18]^, suggesting that silencing of TSPAN1 may be a potential therapeutic target for the treatment of PDAC^[18]^. Further studies in which the expression of MMP2 in isolated CTC is evaluated, in parallel to TSPAN1, are required to confirm the potential role of TSPAN1 in migration of cancer cells via blood to distinct tumor metastatic sites.

Independently of the pathogenic role of TSPAN1, here we evaluated the potential utility of combining anti-TSPAN1 and anti-EpCAM antibodies for the detection and isolation of CTC based on immunomagnetic enrichment, followed by CTC filtration. CAPAN-2 or MIA PaCa-2 cancer cells spiked at known numbers in pre-defined volumes of normal human blood showed a recovery of around two thirds and half the spiked CAPAN-2 and MIA PaCa-2 cells, respectively, both cell lines being found at very low frequencies in the depleted cell fractions. Note that the percentage of recovered spiked cells was notably higher than that observed for the same blood samples and cell lines when they were only stained with the anti-EpCAM reagent but not the anti-TSPAN1 antibody. Altogether, these results support the notion that the immunomagnetic enrichment method used in combination with the anti-TSPAN1 and the anti-EpCAM antibodies might be a good approach for CTC detection in blood of PDAC patients. In line with this hypothesis, further testing of this approach in a small group (*n* = 9) of PDAC patients confirmed the improved CTC recovery, with methods based on simultaneous TSPAN1 and EpCAM staining showing presence of CTC in a significant fraction of the blood samples based on the screening of a relatively limited volume of blood. While Adams *et al*.^[28]^ reported the presence of circulating atypical EpCAM^+^ macrophages (i.e., circulating cancer-associated macrophage-like cells) in blood of both breast and pancreatic cancer patients following enrichment by blood filtration, we did not find CD45^+^ EpCAM^+^ cells in any of the patients here analyzed. Further studies in larger blood volume from larger patient cohorts in comparison with exosome detection^[29-31]^ are required to confirm our preliminary results.

## Declarations

### Copyright

© The Author(s) 2020.
